# Fully automatic acute ischemic lesion segmentation in DWI using convolutional neural networks

**DOI:** 10.1016/j.nicl.2017.06.016

**Published:** 2017-06-13

**Authors:** Liang Chen, Paul Bentley, Daniel Rueckert

**Affiliations:** a BioMedIA Group, Department of Computing, Imperial College London, 180 Queen's Gate, London SW7 2AZ, UK; b Division of Brain Sciences, Department of Medicine, Imperial College London, Fulham Palace Road, London W6 8RF, UK

**Keywords:** Acute ischemic lesion segmentation, DWI, Deep learning, Convolutional neural networks

## Abstract

Stroke is an acute cerebral vascular disease, which is likely to cause long-term disabilities and death. Acute ischemic lesions occur in most stroke patients. These lesions are treatable under accurate diagnosis and treatments. Although diffusion-weighted MR imaging (DWI) is sensitive to these lesions, localizing and quantifying them manually is costly and challenging for clinicians. In this paper, we propose a novel framework to automatically segment stroke lesions in DWI. Our framework consists of two convolutional neural networks (CNNs): one is an ensemble of two DeconvNets (Noh et al., 2015), which is the EDD Net; the second CNN is the multi-scale convolutional label evaluation net (MUSCLE Net), which aims to evaluate the lesions detected by the EDD Net in order to remove potential false positives. To the best of our knowledge, it is the first attempt to solve this problem and using both CNNs achieves very good results. Furthermore, we study the network architectures and key configurations in detail to ensure the best performance. It is validated on a large dataset comprising clinical acquired DW images from 741 subjects. A mean accuracy of Dice coefficient obtained is 0.67 in total. The mean Dice scores based on subjects with only small and large lesions are 0.61 and 0.83, respectively. The lesion detection rate achieved is 0.94.

## Highlights

• A novel framework based on deep CNNs to segment the acute ischemic lesions in DWI.
• It is the first fully automatic method developed for this problem.
• The algorithm is validated on a large real clinical dataset.
• It achieves very good results, which is 0.67 in terms of the Dice coefficient in average.

## 1 Introduction

Stroke is one of the major causes of long-term disability and death globally (Lopez et al., 2006). Cerebral ischemia causes approximately 80% of strokes (Feigin et al., 2003). A number of factors such as energy depletion and cell death are thought to lead to ischemic brain injuries (Dirnagl et al., 1999). Brain imaging is one of the most important methods to assess patients suffering from ischemic stroke (van der Worp and van Gijn, 2007) and computed tomography (CT) and magnetic resonance imaging (MRI) are usually acquired (Latchaw et al., 2009). CT is more widely used because it is faster and less expensive while MRI has much higher sensitivity for the acute ischemic lesions (Lansberg et al., 2000). Particularly, diffusion-weighted MR imaging (DWI) has advantages in diagnosis of acute ischemic lesion in the early stage.

**Fig. 1:**
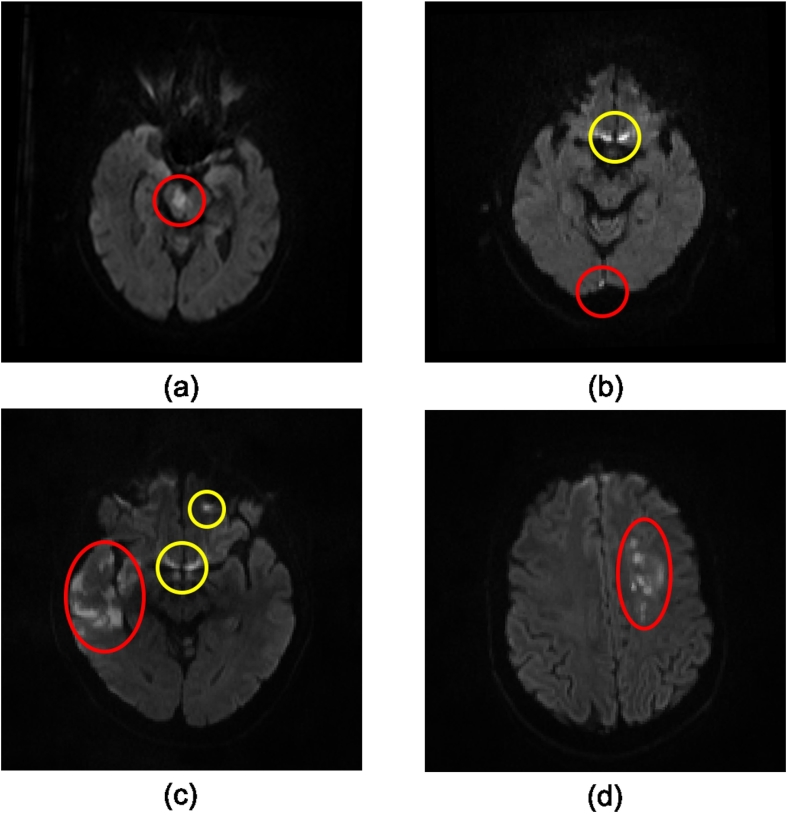
Examples of acute ischemic lesions in DWI. The red circles indicate the acute ischemic lesions and the yellow ones show the artefacts. (For interpretation of the references to color in this figure legend, the reader is referred to the web version of this article.)

The detection and quantification of acute lesions in DWI is important for the diagnosis and treatment of the ischemic stroke. It may allow for accurate estimation of acute lesion volumes. Lesion volume estimation may be important for hyper-acute therapy decision-making, e.g. in determining the ratio of reversible hypo-perfusion to irreversible infarct core (Wouters et al., 2016). Furthermore, acute lesions can be profiled anatomically in terms of volumes of anatomical-functional regions of interest, by superimposing standard atlas-derived or fMRI-derived regions (Rinne et al., 2013). However, manual segmentation of acute ischemic lesions is expensive in terms of time and human expertise. Several automatic and semi-automatic methods have been proposed to assist clinicians to address this problem (Charoensuk et al., 2015, Dwyer et al., 2008, Jacobs et al., 2000, Li et al., 2009, Mah et al., 2014, Martel et al., 1999, Soltanian-Zadeh et al., 2006). A common limitation of these models is that they were developed on small datasets which only contain tens of subjects. Since the ischemic lesions can occur anywhere in the brain in various shapes and sizes (see Fig. 1) (van der Worp and van Gijn, 2007), a small dataset makes it difficult to cover the large variation in position, shape, and size. Most of these algorithms are based on multi-modal MRI including T1-weighted, T2-weighted, fluid attenuation inversion recovery (FLAIR), DWI, and apparent diffusion coefficient (ADC) (Jacobs et al., 2000, Maier et al., 2017). Two of them only based on DWI are semi-automatic: The first one is an adaptive thresholding algorithm incorporating a spatial constraint (Martel et al., 1999). The fully automatic adaptive thresholding segmentation is likely to fail in cases where there are small lesions and/or lesions in low contract to the normal tissue. Therefore, manual editing was introduced to refine the automatic segmentations. The second one is based on active contours algorithms (Charoensuk et al., 2015), where before applying the proposed algorithms, image slices with artefacts are manually removed. In addition, human experts mark bounding boxes around the target lesions to initialize the algorithm. To the best of our knowledge, Mah et al. (2014) proposed the only fully automated method to segment ischemic damage based on a large DWI dataset. However, their approach was dependent on a reference set of normal brain images and it was only applied to lesions in the occipital lobe.

**Fig. 2:**
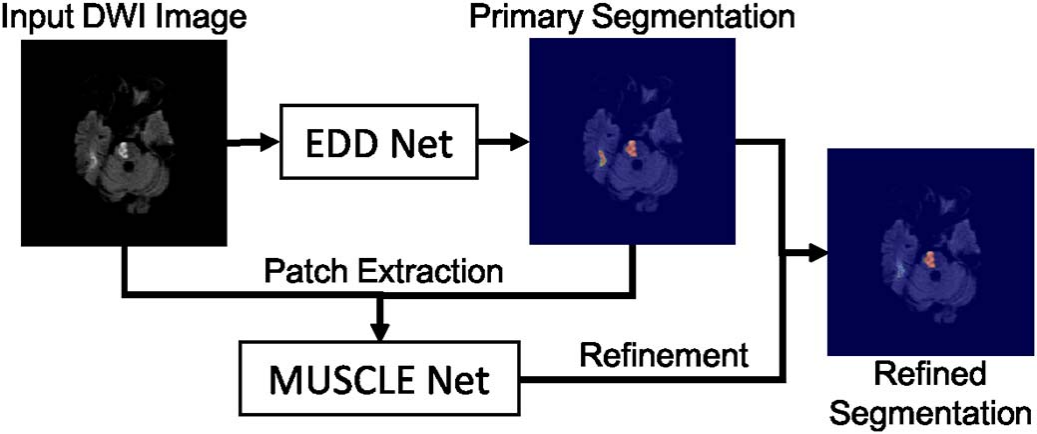
The overview of the proposed CNN based system to segment the acute ischemic lesions in DWI. It comprises the EDD Net and the MUSCLE Net. The EDD Net conducts the semantic segmentation on the input DWI. Based on the output of the EDD Net, patches containing small lesions are extracted and they are evaluated by the MUSCLE Net so that many false positives are removed. The refined segmentation is therefore obtained.

In clinical practice, semi-automatic methods are still too costly and fully automatic algorithms are preferred. Although multi-modal images provide rich information about lesions, pre-processing such as resampling and co-registration are required which can lead to inaccuracies. In this paper, we propose a fully automatic system (Fig. 2) to segment acute ischemic lesions in a large DW image dataset based on deep convolutional neural networks (CNNs). Compared to traditional image analysis algorithms, CNNs have major advantages, including end-to-end training and feature learning (Bengio et al., 2013). Our system consists of two networks, namely the EDD Net and the MUSCLE Net. The EDD Net is an ensemble of two DeconvNets (Noh et al., 2015) and the MUSCLE Net is the MUlti-Scale Convolutional Label Evaluation Net. The input to the proposed system are 2D slices consisting of DWI. The EDD Net firstly outputs a primary segmentation probability map. The binary segmentation obtained by thresholding the probability map contains both lesions and several false positives. The MUSCLE Net re-evaluates all the detections by the EDD Net and excludes many false positives using both the probability map and the original input image.

The acute ischemic lesion segmentation problem is formulated as a semantic segmentation task. However, the task of semantic segmentation of acute ischemic lesions is different from that of objects in natural images. In natural images, the target objects of interest are dominant in images (e.g. images in the PASCAL VOC (Everingham et al., 2015) dataset) while several acute ischemic lesions can be so small (Fig. 1 (b)) that they are easy to be overlooked by observers. In addition, it is also difficult to distinguish the boundaries between ischemic lesions and normal tissue (Fig. 1 (c) and (d)) while objects in natural images are often characterized by sharp edges to the background. Furthermore, there are many artefacts which have similar appearance to the lesions in DWI (Fig. 1 (b) and (c)). Air is one of the main resources of these artefacts. They are the major sources of false positives for automated lesion segmentation techniques.

In this paper, we propose a novel system to address the ischemic lesion segmentation problem. A key contribution is its ability to handle the lesions of various sizes and shapes while minimizing the number of false positives. Our system achieves the state-of-the-art of the ischemic lesion segmentation performance in DWI while being validated on a large clinical dataset from over 700 patients.

## 2 Related work

In this section, we review two categories of related work: First, methods that address the brain tumor segmentation (BRATS) (Menze et al., 2015) and ischemic stroke lesion segmentation (ISLES) (Maier et al., 2017) challenges are reviewed. Secondly, we review several CNN-based segmentation approaches that have been recently introduced into medical imaging.

### 2.1 Brain tumor and lesion segmentation

In the BRATS challenges held in 2016, the dataset contains a number of subjects with gliomas and the task is to develop automatic algorithms to segment the whole tumor, the tumor core and the Gd-enhanced tumor core based on multi-modal MR images. In the latest competition (Menze et al., 2015), over half of the methods were based on deep neural networks and they achieved top results. For instance, the hyperlocal features (original input image) are used prior to the final segmentation to improve the accuracy (Chang, 2016). As a pixel-level segmentation problem, there are much more non-tumor pixels than the ones belong to part of the tumors, which means there is a significant label imbalance. To alleviate the imbalance, Lun and Hsu (2016) proposed a re-weighted loss function. Randhawa et al. (2016) also modified the cross-entropy loss function so that the segmentations at tumor edges could be improved. Instead of analysing multi-modal MRIs in 2D, the DeepMedic approach (Kamnitsas et al., 2016a) performs segmentation of tumors in 3D while using extended residual connections. In addition to deep learning algorithms, machine learning approaches based on the random forests (Ellwaa et al., 2016, Folgoc et al., 2016, Lefkovits et al., 2016, Song et al., 2016) also demonstrate good performance using hand-crafted features.

The segmentation of sub-acute ischemic stroke lesion is one of the tasks in ISLES 2015 (Maier et al., 2017), which attracted many entries. The challenge is to automatically segment sub-acute ischemic stroke lesions based on multi-modal MR images. Compared with the dataset in the BRATS, the dataset used in the ISLES is smaller. Similar to brain tumors, sub-acute ischemic stroke lesions are difficult to segment. In terms of methods proposed, these range from machine learning based methods to deformation based methods. Among the top ranked approaches, DeepMedic (Kamnitsas et al., 2015, Kamnitsas et al., 2016b) was the best, which is a multi-scale 3D CNN with fully connected CRFs achieving a Dice score of 0.59 in testing. The second best performing method used a modified level-set approach embedded with the fuzzy C-means algorithm (Feng et al., 2015) while the third best method is based on random forests and contextual clustering (Halme et al., 2015), which is a typical way of segmenting lesions like those in BRATS. They achieved Dice scores of 0.55 and 0.47, respectively. The Dice scores reported by most other attendees ranged from 0.3 to 0.5.

Most of the successful CNN based methods in both BRATS and ISLES derive a problem specific CNN architecture from generic ones. This is because in medical imaging there is a limited number of images with labels available for training. To explore the distinctive lesion features, specific domain knowledge is still helpful.

### 2.2 Other CNN-based approaches to segmentation

In molecular imaging, a cascaded CNN called deep contour-aware network (DCAN) (Chen et al., 2016) has been shown to be successful in the gland segmentation task. Prior to the final segmentation, a primary gland object segmentation and a gland contour segmentation are produced separately. The final segmentation is then obtained by fusing the object and contour segmentations. The segmentations are based on multi-level contextual features extracted from the fully convolutional layers. In cell segmentation and tracking scenario, the U-Net approach (Çiçek et al., 2016, Ronneberger et al., 2015) performs well. In its architecture, the context and location information of cells are incorporated. Similar to the DeconvNet approach (Noh et al., 2015), the U-Net (Ronneberger et al., 2015) has a series of convolution and deconvolution layers to construct the output based on coarse feature maps. In abdominal imaging, multi-level deep convolutional networks have been proposed to segment the pancreas in CT images (Roth et al., 2015). This uses a hierarchical coarse-to-fine method studying images from patch level to superpixel/region level. In cardiac imaging, a left ventricle segmentation approach for MR images has been proposed that combines deep CNNs and deformable models (Avendi et al., 2016).

Similar to the deep networks proposed for brain lesion segmentation, generic CNN architectures are often customized for many other medical imaging tasks. However, the U-Net (Ronneberger et al., 2015) is a generic architecture which can be easily adapted to other cases in medical imaging. More specifically, it is not a task specific method that requires specific prior knowledge (e.g. the input data has to be homogeneous in 3D). Furthermore, since it is a fully convolutional network, the input is flexible in terms of sizes and dimensionality.

In addition to the U-Net (Çiçek et al., 2016, Ronneberger et al., 2015), the fully convolutional network (Long et al., 2015) and the DeepLab (Chen et al., 2014) are another two generic CNNs for segmentations. The FCN (Long et al., 2015) is the first CNN which allows end-to-end training for the semantic segmentation problem. It inherits the convolution and pooling layers from contemporary CNNs, including the AlexNet (Krizhevsky et al., 2012), the VGG-Net (Simonyan and Zisserman, 2014), and GoogLeNet (Szegedy et al., 2015), in image classification problems. It adapts them into fully convolutional styles for the semantic segmentation task. The FCN (Long et al., 2015) learns features in multiple scales. The DeepLab (Chen et al., 2014) is a type of improvement to the FCN (Long et al., 2015). In order to gain deep features, the FCN (Long et al., 2015) performs many convolutions and poolings which decrease the image resolutions while the DeepLab (Chen et al., 2014) contributes the atrous convolution and atrous spatial pyramid pooling (ASPP) layers which keep the depth of features without decreasing the image resolutions. In ordinary convolutions, features are extracted sparsely while dense features are extracted using the atrous convolutions.

## 3 Our approach

**Fig. 3:**
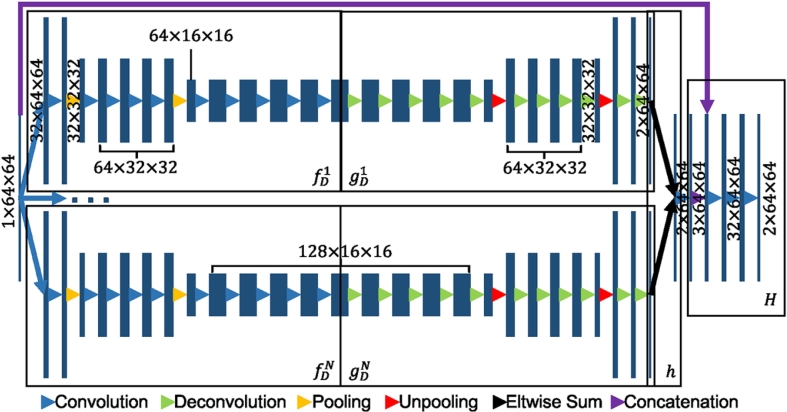
The architecture of the proposed EDD Net. The rectangles in different sizes indicate data blobs in different sizes. The height shows the size of each piece of data, e.g. 64 × 64. The width shows the number of data pieces in each blob, e.g. 1, 32. Arrows in difference colors stand for different operations. (For interpretation of the references to color in this figure legend, the reader is referred to the web version of this article.)

The proposed lesion segmentation framework consists of two modules: The first one is an ensemble of *N* adapted DeconvNets (Noh et al., 2015) (EDD Net) (Fig. 3) and the second one is a MUlti-Scale Convolutional Label Evaluation Net (MUSCLE Net) (Fig. 5). While the EDD Net attempts to achieve optimal lesion segmentation at lesions in all scales, the MUSCLE Net focuses on lesions that have been detected at small scales and aims to remove false positives.

### 3.1 EDD Net

Fig. 3 shows the architecture of the proposed EDD Net. The input is an image patch, which is fed into *N* parallel DeconvNets (Noh et al., 2015) to infer the semantic segmentations respectively. The results from both are then combined. The combination is concatenated with the input image patch. Several convolution layers are added in the end to produce the final output.

The basis CNN architecture, i.e. the DeconvNet (Noh et al., 2015) is selected among several generic CNN architectures for semantic segmentation, including the U-Net (Ronneberger et al., 2015), DeepLab (Chen et al., 2014) and the FCNs (Long et al., 2015). The basis network has a stack of convolution and pooling layers in the convolution stage and a stack of corresponding deconvolution and unpooling layers in the deconvolution stage. Within each stack, there are several convolution/deconvolution layers. Between two stacks, there is a pooling/unpooling layer. The number of stacks and the number of layers in each stack define the size of the network. The proposed basis network has three stacks of convolution layers and two pooling layers in the convolution stage, which leads to the best results.

In segmentation, contextual information often contributes important knowledge to solve the label assignment. However, the appropriate level of contextual information is often difficult to identify. Excessive amounts of context can hinder the segmentation of lesions and insufficient context makes it difficult to distinguish between lesions and artefacts. If the network grows deep, i.e. has many convolution and pooling layers, it processes a large amount of contextual information. However, with the increasing number of convolution and pooling layers, the input is down-sampled further and further and therefore the resulting feature maps have lower and lower resolutions. In this case, small lesions are gradually eliminated by subsequent down-sampling steps and it can be difficult to reconstruct these. In contrast, if the network is shallow, i.e. using only few convolution and pooling layers, only limited context is used. In this case, lesions and artefacts may have similar feature representations making it difficult for the classifier to distinguish between them.

In our approach, we propose to use image patches instead of image slices as the input. This has three major advantages: Firstly, it modifies the data distribution. For a given image slice, there is a significant imbalance between pixels that represent normal tissues compared to those of lesions since acute ischemic lesions occur locally (Dirnagl et al., 1999). The signals representing lesions are as weak as those representing noise and artefacts among the whole data distribution. However, the lesion signals can be apparent among the data distribution based on image patches. Secondly, a large number of patches can be extracted from image slices, which is a fundamental requirement for CNN training. In contrast, if the training data is based on image slices, there is only limited number of candidates available. Finally, as image patches are smaller than image slices, the batch size in training can be larger, which makes the training more efficient.

We propose to adopt the DeconvNet (Noh et al., 2015) as the basis network of the EDD Net. In addition to convolution and pooling layers, the DeconvNet (Noh et al., 2015) has corresponding deconvolution and unpooling layers to create the segmentation probability map from the coarse feature maps. For the input image patch **x**, assume $\tilde{x}$ is the feature maps obtained from the convolution and pooling operations. *f*(⋅) and *g*(⋅) are the convolution and deconvolution functions which jointly produce the segmentation map **y**, i.e.

$$\tilde{x}=f(x),y=g(\tilde{x}).$$

In different architectures, the *f*(⋅) functions are similar, which is the composition of several convolutions and poolings, while different strategies are usually used in *g*(⋅).

In the DeepLab approach (Chen et al., 2014), the *g*(⋅) function is a bilinear interpolation function upsampling the coarse feature map into the segmentation map directly. In the FCN approach (Long et al., 2015), the *g*(⋅) not only bilinearly upsamples the feature map but also fuses it with the feature maps obtained at higher resolutions as these contain more image details. Therefore, more small lesions are detected. However, they are difficult to distinguish from artefacts.

In the U-Net (Ronneberger et al., 2015), the *g*(⋅) is modelled in a more sophisticated and powerful fashion. Here, the final segmentation is constructed step by step. In each step, the feature map is upsampled to a higher resolution first, which corresponds to a pooling layer before. The upsampled feature maps are then concatenated with the feature maps before the corresponding pooling layer. Afterwards, a few layers of convolutions are performed on the concatenation. As a result, the segmentation obtained from the U-Net (Ronneberger et al., 2015) has less false positives than that from the FCN (Long et al., 2015) since these convolutions detect and eliminate several false positives.

**Fig. 4:**
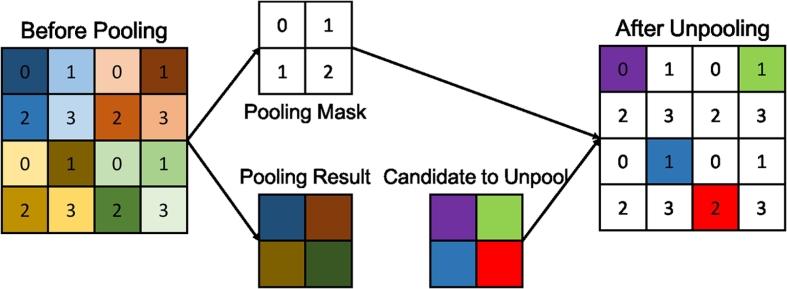
The max pooling and unpooling strategy demonstrated in the DeconvNet approach (Noh et al., 2015). In the pooling stage, the position of the maximum activation is recorded within each filter window by a mask. In the unpooling stage, the entries are placed in the unpooled map according to the mask.

In the DeconvNet approach (Noh et al., 2015), there are additional pooling masks **m** (Fig. 4) output from pooling layers who record the locations of the maximal activations. Thus, the specific functions in the DeconvNet (Noh et al., 2015) can be written as:

$$\tilde{x},m=f_{D}(x),y=g_{D}(\tilde{x},m).$$

The *g*_*D*_(⋅) function represents the deconvolution and unpooling operations. The pooling masks **m** are used for upsampling so that the semantic output can be better constructed. Similar to the U-Net (Ronneberger et al., 2015), the DeconvNet (Noh et al., 2015) employs a number of deconvolution layers to construct the output step by step, which results in accurate segmentations. In contrast, the U-Net (Ronneberger et al., 2015) uses feature maps before pooling layers to assist recovering image details, however, this can introduce artefacts and noise. Instead, the pooling masks used in the DeconvNet approach (Noh et al., 2015) exclude the artefacts and noise.

We propose to combine *N* DeconvNets (Noh et al., 2015) to produce an ensemble of classifiers in order to further enhance the results. Let *h*(⋅) be the ensemble function fusing the *N* networks together, i.e.

(1) $$h(x)=g_{D}^{1}(f_{D}^{1}(x))⊕g_{D}^{2}(f_{D}^{2}(x))⊕\cdots ⊕g_{D}^{N}(f_{D}^{N}(x)).$$

Since the *N* DeconvNets (Noh et al., 2015) are initialized differently, they converge at different optima but all of them are able to produce accurate lesion segmentations. An ensemble of all CNNs therefore benefits for performance improvement because of their accuracy and diversity (Zhou, 2012).

Furthermore, inspired by the U-Net (Ronneberger et al., 2015) we propose additional convolution layers at the end of the naive ensemble to refine the segmentation. There are many convolutions and deconvolutions between the original input image and the semantic segmentation. The network may eliminate some details in the input image during the feed-forward pass. We propose to concatenate the input image and the segmentation probability map as well as to add a few convolution layers so that the segmentation can be refined according to the original image. The refinement yields marginal increase of performance. Therefore, the function that the proposed EDD Net performs is

(2) H(x)=r(h(x),x).

Here *r*(⋅,⋅) performs the concatenation and convolutions after the naive ensemble. The loss function of the EDD Net is therefore

(3) $$\begin{array}{c} \ell =\lambda_{1}\ell_{1}(H(x),y)+\lambda_{2}\ell_{2}(h(x),y)+\lambda_{3}\ell_{3}(g_{D}^{1}(f_{D}^{1}(x)),y)\\ \;+\lambda_{4}\ell_{4}(g_{D}^{2}(f_{D}^{2}(x)),y)+\cdots +\lambda_{N+2}\ell_{N+2}(g_{D}^{N}(f_{D}^{N}(x)),y). \end{array}$$

In the loss function, *ℓ*_*i*_(*i* = 1,2,…,*N* + 2) is the cross-entropy loss function and the *λ*_*i*_ is the corresponding weight. The loss function is optimised via back-propagation as usual.

The EDD Net is a fully convolutional network since all of its subnets are fully convolutional. Therefore, the size of the input image patch is flexible. In practice, we use the image patches to train the network and we test it on the whole image slice.

### 3.2 MUSCLE Net

The EDD Net identifies many acute ischemic lesions correctly. However, it also produces many false positive clusters (i.e., aggregation of voxels) which have similar appearance with the small lesions. To remove them, we propose a second network, called MUSCLE Net, which evaluates the labels of small lesions detected by the EDD Net in order to differentiate between false and true positives.

**Fig. 5:**
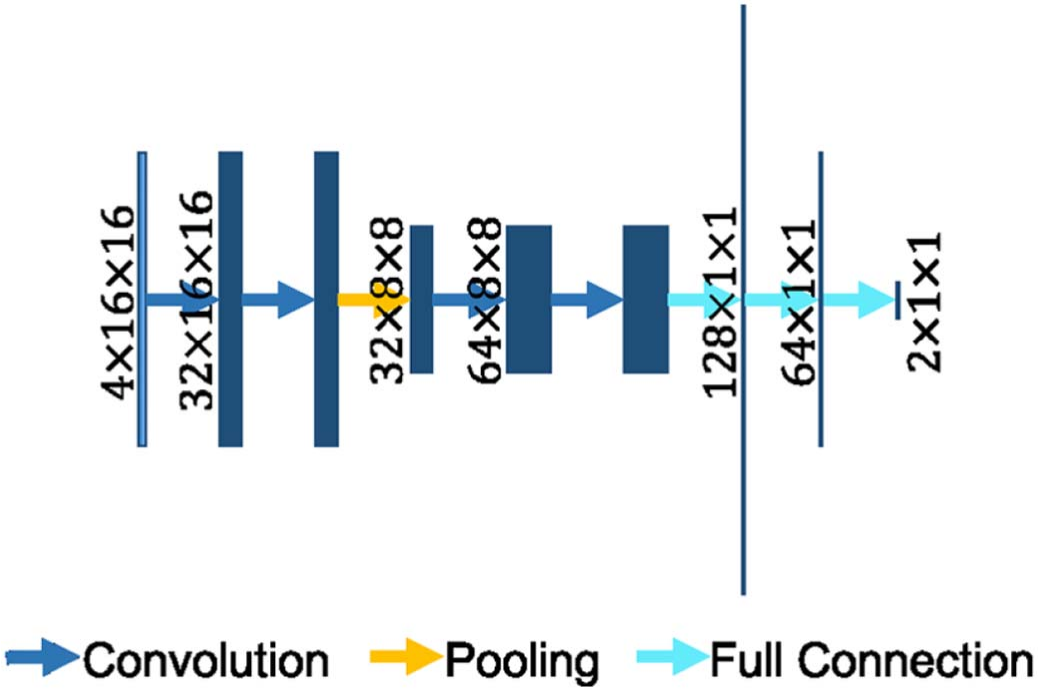
The architecture of the MUSCLE Net. The rectangles stand for the data blobs. Their heights represent the sizes of data pieces, e.g. 16 × 16. Their widths show the number of data pieces in the blobs, e.g. 4, 32. In the fully connected layers, the lengths of strings demonstrate the number of elements in the layers. Arrows in different colors show different operations. (For interpretation of the references to color in this figure legend, the reader is referred to the web version of this article.)

The architecture of the MUSCLE Net is shown in Fig. 5. The input is a stack of image patches at three scales extracted from the original DWI as well as the probabilistic output from the EDD Net. The MUSCLE Net aims at evaluating if the candidate is a real lesion or not. Considering the input patches are fairly small, the MUSCLE Net has limited convolutional layers.

The architecture of the MUSCLE Net is based on a mini VGG-Net (Simonyan and Zisserman, 2014). It focuses on small lesions locally so that the input image patches are relatively small. The MUSCLE Net consists of four convolution layers, one pooling layer, and three fully connected layers. The convolution and pooling layers extract the distinctive features from the input and the fully connected layers act as a classifier.

**Fig. 6:**
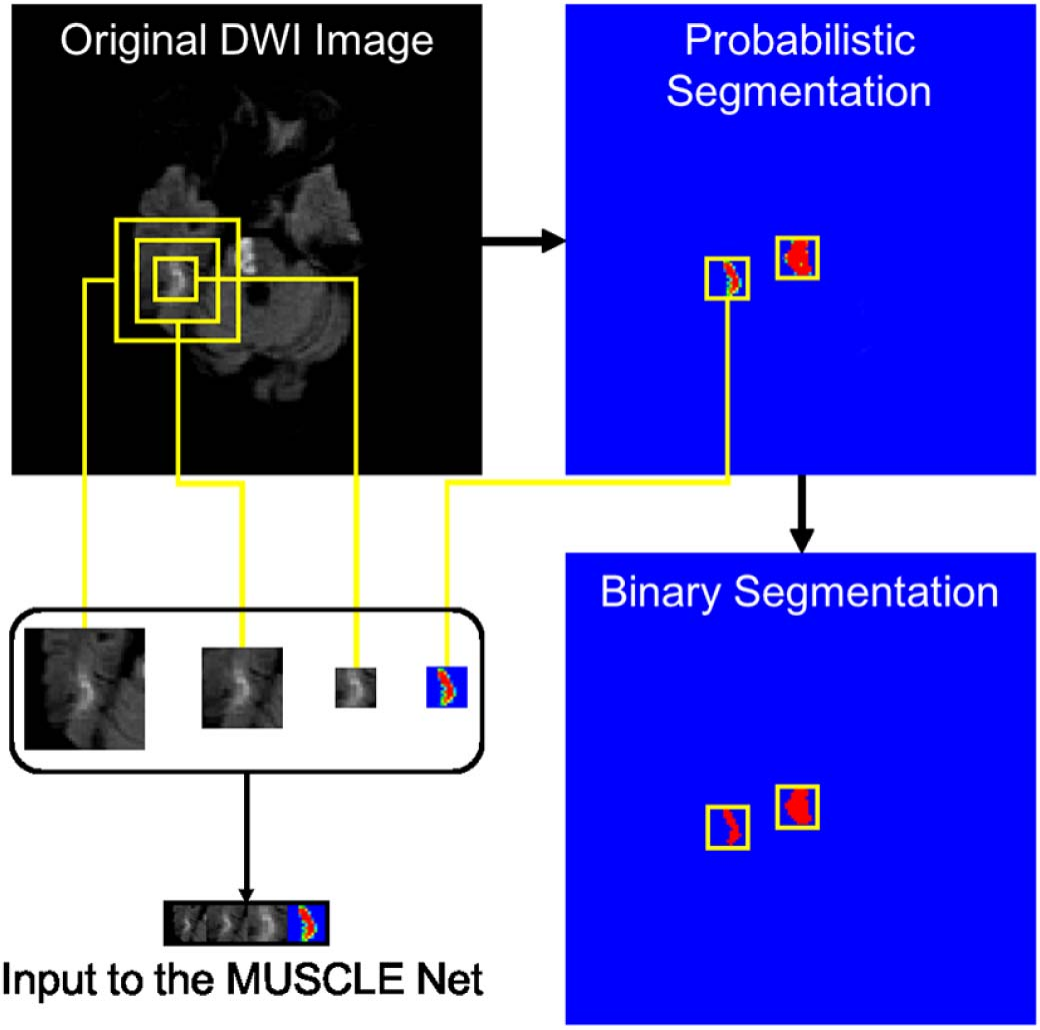
The derivation of the input to the MUSCLE Net. The probabilistic segmentation is obtained from the EDD Net. The binary segmentation is obtained by thresholding the probabilistic segmentation. Candidate small blobs are detected in the binary segmentation. The corresponding patches are extracted in the original DWI in multiple scales and the probabilistic segmentation map. They are then resized and concatenated resulting in the input to the MUSCLE Net.

The input patch set is derived as follows: First, the primary binary lesion segmentation map is obtained by thresholding the probabilistic segmentation map which is the output of the EDD Net. Based on the binary segmentation map, small candidate lesions are detected using connected-component analysis. Original image patches at multiple scales are extracted around them, as well as the corresponding probabilistic segmentation as computed by the EDD Net. This procedure is described in Fig. 6. The real lesions (true positives) are labelled as positive instances while the false positives are labelled as negative ones.

The MUSCLE Net outputs results in instance level rather than pixel level, which are the probabilities of the candidates being lesions. They are then fused with the pixel level probabilities given by the EDD Net using Bayes' theorem. The final semantic segmentation result is therefore achieved. The loss function used here is the cross-entropy function and it is optimised using the back-propagation algorithm.

### 3.3 Evaluation methods

We propose a number of criteria to evaluate our method. First, the Dice coefficient is used to compare the agreement with manual segmentation. It measures the overlap between the candidate segmentation *X* and the reference segmentation *Y* and is defined as

$$Dice(X,Y)=\frac{2|X\cap Y|}{\left|X|+|Y\right|}.$$

|⋅| denotes the number of pixels in the set. However, the Dice similarity measurement based on overlaps is not robust in all cases: For example, an error of 1 pixel may not affect the Dice coefficient significantly if the ground truth contains hundreds of pixels; however it makes a significant difference where the ground truth is small and only contains a few pixels. Therefore, the average number (m#) and the average pixel-size (mS) of the false positives (FP) and false negatives (FN) are introduced as additional metrics. Our goal is to decrease the number and size of the FP and the FN. In addition, we define the detection rate (DR) as

$$DR=\frac{N_{TP}}{N}$$

where the *N* denotes the number of all subjects and the *N*_*TP*_ denotes the number of subjects with any true positive (TP) lesion detections. Since the FP may mislead clinicians, the DR is expected to be as high as possible.

### 3.4 Implementation details

The CNNs in this paper are implemented using the Caffe framework (Jia et al., 2014). The optimisation during training is achieved using the standard stochastic gradient descent algorithm. The learning rate is fixed as 0.05. The momentum and the weight decay is set to 0.9 and 0, respectively. The weights in networks are initialized using the xavier algorithm (Glorot and Bengio, 2010). The filter size of the convolution and deconvolution layers are 3 × 3 and the stride is 1. The batch normalization technique (Ioffe and Szegedy, 2015) is used. We have limited computation resources and therefore set *N* = 2. In the Eq. (3), we set *λ*_*i*_ = 1,*i* = 1,2,…,*N* + 2.

## 4 Data

### 4.1 Dataset and pre-processing

In this study, DWI scans from 741 acute stroke patients were collected from local hospitals. All clinical images were collected from a retrospective database and anonymized prior to use by researchers. Ethical approval was granted by Imperial College Joint Regulatory Office. The scans were obtained from three different scanners (Siemens) with the following acquisition parameters: field strength: 1.5–3 T; slice thickness: 5 mm; slice spacing: 1.0–1.5 mm; pixel size in x–y plane: 1.40 × 1.40 or 1.80 × 1.80 mm; matrix size: (19–23) ×(128 × 128) or (192 × 192); field of view: 230 × 230 or 267 × 267; echo time 90–93 ms; repetition time 3200–4600 ms; flip angle 90°; phase encoding steps: 95–145. Patients information can be found in Table 1. In all images, the acute ischemic lesions were annotated by experienced experts. We use 380 of them to train and validate our CNNs and the remaining 361 ones are used for testing only. Among the developing images, 274 of them are used for training and 106 ones consist of the validation set.

**Table 1:** Patients information in statistics.

Age (years)	Mean: 68.01, std: 14.8, range: 26–93

Gender (male %)	56.28
Interval from acute clinical presentation to MRI (days)	Median: 2, std: 1.78, range: 0–9
Admission functional severity (NIHSS)	Median: 5, range: 1–30

Since the images were acquired from different scanners under different protocols, several pre-processing steps are performed before experiments. Considering the images are anisotropic in the axial direction (or z-axis) and the resampling is likely to introduce interpolation errors, we will perform analysis of 2D slices instead of 3D volumes. To make sure each pixel in 2D slices has uniform physical pixel size (in mm^2^), homogeneous linear resampling is performed in 2D. All images are resampled to uniform pixel size in 2D of 1.6 mm ×1.6 mm. Subsequently, the intensity distribution of each image is normalized into that of zero mean and unit variance.

### 4.2 Data augmentation

Each DWI scan has a limited number of lesions, if the training data is generated in the image slice level or lesion instance level, there is only a small number of images (patches) available. As CNNs have a large number of parameters and it is necessary to generate a large number of images (patches) to train the CNN. For this, data augmentation is implemented in several ways to produce more training data based on the limited number of DWI: First, extracted images (patches) are horizontally flipped and randomly rotated. Second, the patch extraction strategy also represents a way of data augmentation. It is used to reduce the redundant contextual information and balance the number of normal and lesion pixels but it is an effective way of data augmentation. We sample all pixels labelled as part of lesions. For each of these pixels, we extract a patch around it. That pixel is placed in a random position in the patch. As a result, each patch contains pixels belonging to both lesions and tissues/background in general. If the pixel locates in the center of a very large lesion, the patch extracted based on it may contain pixels only belonging to lesions. A pixel cluster of lesions usually have a number of pixels (e.g. 20). That number of patches (i.e. 20) can be generated.

## 5 Experiments and results

### 5.1 Baseline architectures

Although the DeconvNet (Noh et al., 2015) was selected as the basis CNN in the proposed EDD Net, other generic CNN architectures, including the U-Net (Ronneberger et al., 2015), the DeepLab (Chen et al., 2014) and the FCN (Long et al., 2015), aiming at image segmentation were used as baseline comparison. In this set of experiments, comparisons were among single networks rather than ensembles. The training inputs to all CNNs were patches from the DWI of 64 × 64 pixel size. This was the best patch size for this task (see Section 5.2). Since each architecture had its own characteristics, it was difficult to adapt them so that they had exactly the same size of the receptive field. Fortunately, our results in Section 5.2 showed the performance was robust to the size of the receptive field when the image patch size was 64 × 64. When adapting the candidate CNN architectures into our dataset, we preserved their key features. More specifically, the adapted DeepLab (Chen et al., 2014) contained atrous convolution and atrous spatial pyramid pooling (ASPP) layers. The adapted FCN (Long et al., 2015) was still in the fully convolutional configurations and used a multi-scale approach. The adapted U-Net (Ronneberger et al., 2015) had concatenations between related layers. The adapted DeconvNet (Noh et al., 2015) retained the featured unpooling layer. No post-processing operations such as the CRFs were used in any architecture.

The results were displayed in Table 2. All CNNs shared very high detection rates. The DeconvNet (Noh et al., 2015) clearly outperformed the other approaches. Since the gap between the U-Net (Ronneberger et al., 2015) and the DeconvNet (Noh et al., 2015) was not very significant, we performed paired *t*-test between them in the testing dataset. The *p*-value is 1.12 × 10^ −4^, which indicated that the DeconvNet (Noh et al., 2015) was superior to the U-Net (Ronneberger et al., 2015) in this case. As they share similar *f*(⋅) functions, the key lies in the *g*(⋅) functions. In the *f*(⋅) functions, many convolution and pooling operations are performed, which diminishes the activations of lesions in small scales. Basically, all architectures except the DeconvNet (Noh et al., 2015) employ the bilinear interpolation strategy to upsample the coarse feature maps. This bilinear interpolation makes it difficult to reconstruct the small lesions based on the weak activations. The DeepLab approach (Chen et al., 2014) produces the output by conducting the bilinear interpolation on the feature maps in the lowest resolution, which introduces many false negatives. The FCN approach (Long et al., 2015) combines feature maps at multiple resolutions to construct the segmentation map. The feature maps in high resolutions contain signals from small lesions but artefacts and noise as well, which results in a large number of false positives in average. The U-Net (Ronneberger et al., 2015) is equipped with more powerful operations in its *g*(⋅) function so that it performs better than the former two networks. The success of the DeconvNet (Noh et al., 2015) in this case is due to the recorded pooling masks and the unpooling strategy. They work jointly and are able to preserve the signals from small lesions. Despite that the activations of small lesions are weakened, if they are recorded by the pooling masks, they are likely to be reconstructed in the deconvolution stage. In summary, the pooling mask recording and unpooling strategy works better than bilinear interpolation when there are small lesions.

**Table 2:** Performance of the baseline CNN architectures. In each measurement, results on the training, validation, and testing datasets are reported respectively. The DeconvNet (Noh et al., 2015) is superior to the others in most measurements. In each row, the bold number indicates the most significant performance.

Architecture		DeepLab without CRF (Chen et al., 2014)	FCN (Long et al., 2015)	U-Net (Ronneberger et al., 2015)	DeconvNet (Noh et al., 2015)
Side length of receptive field		44	52	46	44
Dice	train	0.60	0.66	**0.71**	**0.71**
	val	0.55	0.60	**0.64**	0.62
	test	0.48	0.50	0.52	**0.55**
m#FP	train	10.35	11.73	**7.86**	8.32
	val	11.51	13.30	**8.95**	10.08
	test	12.81	16.44	12.85	**11.78**
m#FN	train	4.80	2.96	2.35	**2.19**
	val	4.91	4.00	**3.92**	4.03
	test	5.22	**3.88**	3.99	3.99
mSFP	train	**7.23**	8.40	9.56	8.60
	val	**7.29**	8.66	9.10	8.69
	test	**8.25**	9.92	11.50	10.14
mSFN	train	3.34	2.03	2.17	**1.80**
	val	6.53	5.84	6.20	**5.11**
	test	4.08	3.66	4.17	**3.58**
DR	train	0.97	**0.99**	**0.99**	**0.99**
	val	0.98	**0.99**	**0.99**	0.97
	test	0.93	**0.94**	**0.94**	**0.94**

### 5.2 Patch size and receptive field

The DeconvNet (Noh et al., 2015) has been validated that it is the best baseline architecture among all candidate CNN architectures. In addition to the CNN architecture, the configuration of the network influences the performance significantly. It is mainly in two aspects which are the size of the input image patches and that of the network's receptive field. As mentioned before, the size of image patches in the training stage determines the data distribution. The size of the network's receptive field determines the amount of contextual information being considered. They work jointly and experiments in this section aim at discovering how do they affect the CNN's performance.

Single DeconvNets were used in the following experiments. In terms of the input patches, four different sizes were tested. The maximum was the whole image slice. The different sizes of the receptive fields were realized by employing different numbers of convolution and pooling layers. For instance, each DeconvNet branch in the EDD Net (Fig. 3) had the receptive field in 64 × 64 pixels.

Table 3 displayed the results of the DeconvNets (Noh et al., 2015) for different configurations. It was obvious that when the input patches in the training stage were small in size (32 × 32) or large (i.e. the full image size 128 × 128), the CNN could not perform well in the semantic segmentation task since they contained either insufficient or excessive contextual information. Although small patches could help discriminate the lesions from the normal tissue, which reduced the false negatives to the minimum, it was difficult for the network to distinguish between artefacts and the real lesions. As a result, there was a large number of false positives introduced. In the other extreme case where the input was the full image slice, small objects including artefacts and lesions were easily eliminated by the numerous convolutions and poolings. Therefore, few false positives were introduced but there were more false negatives. In the mean time, many true positives were ignored by the CNN so that the detection rate fell down. Not surprisingly patches of medium sizes (64 × 64 and 96 × 96) were able to achieve the trade-off between the numbers of false positives and false negatives and thus the Dice coefficients on the whole increased to reach an optimum.

**Table 3:** Results of the DeconvNet (Noh et al., 2015) in different configurations. In each measurement, results on the training, validation, and testing datasets are reported respectively. It is clear that the size of training patch size influences on the performance more than the size of network's receptive field. In each row, the bold number indicates the most significant performance.

Size of input patch		32 × 32		64 × 64			96 × 96			128 × 128		
Side length of receptive field		18	32	32	44	64	44	64	96	64	96	128
Dice	train	0.48	0.49	0.71	0.71	**0.74**	0.72	0.69	0.68	0.62	0.63	0.61
	val	0.44	0.44	**0.64**	0.62	**0.64**	0.63	0.59	0.58	0.50	0.53	0.51
	test	0.36	0.36	0.55	0.55	**0.58**	0.54	0.52	0.51	0.47	0.48	0.47
m#FP	train	44.32	38.16	9.09	8.32	5.41	8.53	9.69	12.93	1.68	1.82	**0.96**
	val	43.14	38.96	11.04	10.08	7.88	11.26	12.90	16.08	2.75	2.64	**1.63**
	test	51.23	41.07	12.82	11.78	7.92	13.74	13.18	17.39	3.45	3.41	**1.75**
m#FN	train	2.74	2.63	2.62	2.19	2.12	2.35	**1.93**	1.97	5.40	5.33	5.59
	val	**3.17**	3.41	3.97	4.03	4.39	4.09	4.50	4.41	6.37	6.19	6.52
	test	**2.82**	3.31	3.82	3.99	4.25	3.95	4.26	4.14	6.53	6.41	6.83
mSFP	train	9.34	10.42	6.97	8.60	9.30	7.05	8.73	7.37	3.25	5.10	**2.97**
	val	9.73	10.20	6.51	8.69	8.52	7.29	8.37	7.20	4.07	5.66	**3.10**
	test	10.41	11.30	8.05	10.14	10.63	7.79	9.81	8.01	4.81	6.34	**4.40**
mSFN	train	2.17	2.49	2.21	1.80	1.99	1.99	1.64	**1.57**	3.19	3.01	3.33
	val	4.12	**3.53**	6.67	5.11	7.48	6.00	5.46	6.44	8.18	7.94	8.38
	test	**3.02**	3.47	4.05	3.58	3.70	3.77	3.94	3.53	5.54	5.23	6.22
DR	train	**0.99**	0.98	**0.99**	**0.99**	0.98	**0.99**	**0.99**	**0.99**	0.98	0.98	0.97
	val	**0.99**	**0.99**	**0.99**	0.97	**0.99**	**0.99**	**0.99**	0.98	0.96	0.95	0.95
	test	**0.95**	0.94	0.94	0.94	0.94	0.94	0.93	0.94	0.90	0.91	0.91

It was interesting that the DeconvNets (Noh et al., 2015) were generally robust to the size of the receptive fields in terms of the Dice coefficient when the size of the training input patches was fixed. Particularly when the patch size was extremely small or large, the overall results were stable in terms of Dice coefficient. In these cases, the size difference of the receptive fields was reflected in the number of false positives and false negatives. If the patches were in medium sizes, the Dice coefficient showed little fluctuations. For instance, when the training patches were in 64 × 64 pixels, the networks performed similarly whose receptive fields were in 32 × 32 and 44 × 44 pixels. However, the performance slightly improved when the size of the receptive field increased to 64 × 64 pixels. When the training patches were in 96 × 96 pixels, the DeconvNet (Noh et al., 2015) with the receptive field in 44 × 44 pixels had a slightly better performance compared to those with larger receptive fields.

According to the results, the configuration providing the best performance was chosen as the basis network of the EDD Net. More precisely, the training patches were in 64 × 64 pixel-size and the same as the receptive field. In summary, the training patch size affects the networks' performance more than the receptive field. Patches of medium sizes are preferable. Once the size of training patches is fixed, the network is fairly robust to the size of the receptive field.

### 5.3 Ensemble and refinement

To further improve the performance, the EDD Net was developed based on the DeconvNets (Noh et al., 2015) under the best configuration. Table 4 displayed the results in detail. First, the two DeconvNets (Noh et al., 2015) both provided accurate segmentations as before. Note that the Dice coefficient of them in this experiment were 0.56 which is slightly lower than it in Table 3. It is the fact that training two networks simultaneously is more difficult than a single one as the number of parameters doubles. Therefore, the loss function is more difficult to optimise. Second, it was obvious that the naive ensemble of the two networks led to a significant improvement. This is due to a sharp reduction of the false positives, which results from the diversity of the two DeconvNets (Noh et al., 2015). As both of them have detected most of the lesions, the diversity indicates false positives given by them are different. Fusing them together should be able to decrease a substantial number of false positives.

**Table 4:** Results of the EDD and the MUSCLE Nets. In each measurement, results on the training, validation, and testing datasets are reported respectively. The ensemble contributes a significant improvement to the whole performance. The MUSCLE Net shows its advantage in removing false positives to boost the performance tremendously again. In each row, the bold number indicates the most significant performance. In the rows where all perform the same, no bold numbers are identified.

		DeconvNet 1	DeconvNet 2	Naive ensemble	EDD Net	EDD + MUSCLE Net
Dice	train	0.74	0.72	0.79	0.80	**0.88**
	val	0.64	0.61	0.68	0.69	**0.73**
	test	0.56	0.56	0.62	0.63	**0.67**
m#FP	train	6.82	9.49	4.20	3.78	**0.64**
	val	9.23	12.27	6.33	5.67	**3.14**
	test	10.18	13.38	6.68	5.89	**3.27**
m#FN	train	1.80	1.59	1.51	1.45	**1.45**
	val	4.08	**3.80**	4.02	4.01	4.16
	test	4.02	**3.66**	3.81	3.82	4.07
mSFP	train	8.39	**6.89**	9.55	9.49	8.81
	val	8.09	**7.33**	9.01	8.87	8.95
	test	9.55	**7.37**	10.31	10.53	12.16
mSFN	train	1.86	**1.40**	1.41	1.42	1.42
	val	**5.58**	5.71	5.65	5.62	6.32
	test	3.81	**3.19**	3.49	3.64	4.16
DR	train	0.99	0.99	0.99	0.99	0.99
	val	0.99	0.99	0.99	0.99	0.99
	test	0.94	0.94	0.94	0.94	0.94

Finally, a few convolution layers were added to refine the segmentation provided by the naive ensemble. The naive ensemble of the two DeconvNets (Noh et al., 2015) was so deep that the input patches were likely to lose details when being fed forward. Inspired by the U-Net approach (Ronneberger et al., 2015), concatenating the original input and the result given by the naive ensemble and adding a few convolution layers yielded a refined segmentation. In summary, the ensemble based on the accuracy and diversity of sub-nets makes a significant improvement to the network performance entirely.

### 5.4 The MUSCLE Net

**Fig. 7:**
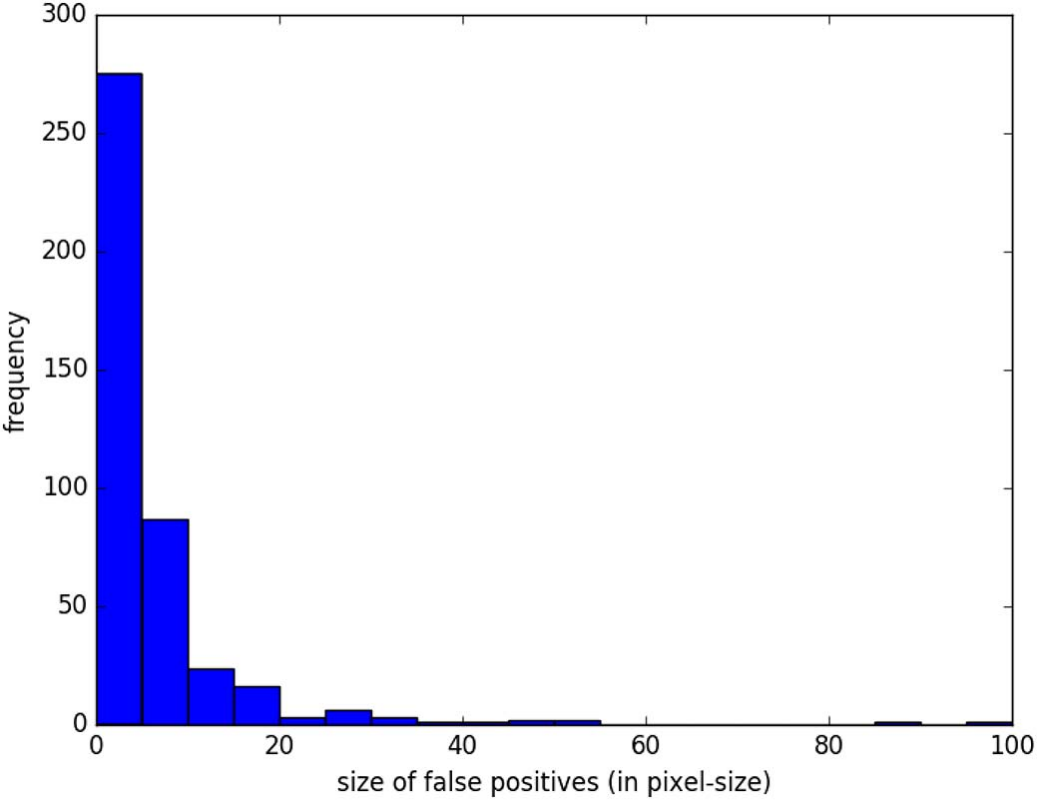
The statistics of the false positives on the validation dataset provided by the EDD Net.

The EDD Net has advantages to segment the acute ischemic lesions in DWI. However, false positives are difficult to avoid. We validated the trained EDD Net on the validation dataset and reported the false positives in Fig. 7. Approximately 99% false positives were of size 60 pixels or less. According to the Table 4, the false positives on the validation dataset were in 8.87 pixels in size on average. Therefore, the MUSCLE Net is only needed to assess candidates within 60 pixels or less in size, which is defined as small objects.

**Fig. 8:**
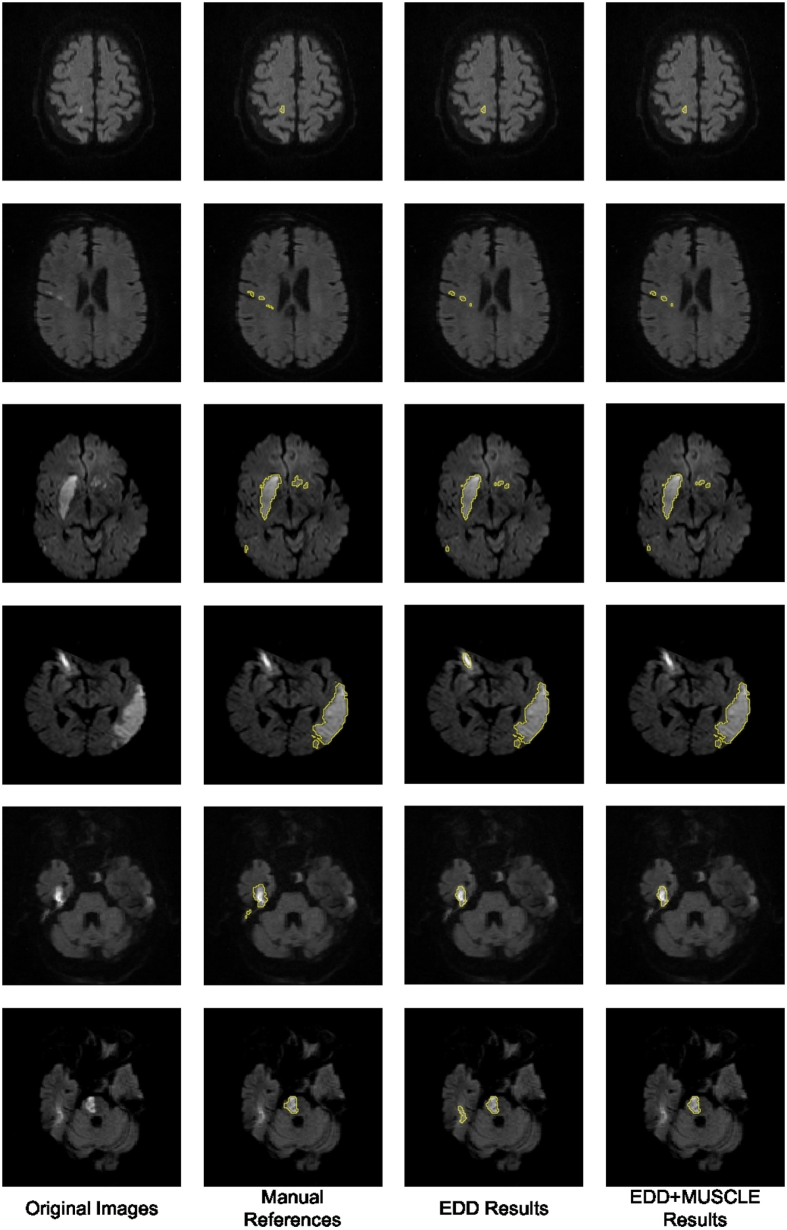
The results of the proposed method. The first column shows the original DWI. The second column displays the manual annotations of the acute ischemic lesions. The third column demonstrates the results given by the EDD Net. The last column illustrates the lesion segmentations refined by the MUSCLE Net.

Table 4 also showed the results of the EDD + MUSCLE Nets. The MUSCLE Net eliminated a large number of false positives without erasing many true positives, which benefited further improvement in performance. According to our observations, the false positives normally appeared isolated without overlap with other lesions. Examples were shown in Fig. 6, Fig. 8. This should be one of the major reasons leading to the success of the label evaluation. Although false positives were removed, their mean size grew, which indicated that most false positives within a few pixel-size were eliminated while some slightly larger ones were remaining. The limitation of the MUSCLE Net is that it is not possible to be integrated with the EDD Net to enable the end-to-end training since the training data generation operation is not differentiable. In summary, the MUSCLE Net is powerful to remove false positives without introducing many false negatives.

### 5.5 Small and large lesions

Apart from the analysis based on the whole testing dataset, it was also interesting to study the performance of our proposed CNNs on datasets with only small or large lesions. First, we computed the mean size of lesions of each subject in our testing dataset and took an average across all subjects. As a result, the mean average size of lesions of the testing subjects was 36.21 pixel-size. Therefore, we regarded subjects with average lesions smaller than 37 pixel-size as the ones with small lesions; otherwise with large lesions. Second, the testing dataset was separated into two subsets: one contained subjects with small lesions and the other one consisted of subjects with large lesions. The former subset had 271 subjects and the latter one had 90 subjects. Third, we evaluated our baseline CNN architectures and proposed EDD and MUSCLE Nets based on the two subsets.

Results were displayed in Table 5. Not surprisingly, the performance of all CNNs dropped down when there were only small lesions. When there were only large lesions, the detection rates were 100%. However, the EDD Net performed significantly better than any of the baseline CNNs. Its mean Dice score was 9% higher than the best baseline CNN. This improvement came from the significant reduction of the number of false positives as its m#FN, mSFP, and mSFN were similar to the baselines'. In addition, the MUSCLE Net further removed nearly half of the false positive artefacts. Importantly, the m#FN of the MUSCLE Net only increased a bit compared to the EDD Net, which indicated that it maintained most of the true positive lesions. In terms of the subjects with large lesions, the Dice score achieved by the EDD Net reached 83%. In this condition, although the MUSCLE Net was still able to remove some small false positives, it could not reflected on the Dice score. The detection rates indicated that when there were large lesions, they can never be ignored by our CNNs. The proposed CNNs might only ignore a few small lesions.

**Table 5:** Performance comparison among adapted existing CNNs and our proposed CNNs on two subsets of testing dataset. One subset consisted of 271 subjects with small lesions and the other one contained 90 subjects with large lesions. The results showed the EDD Net performed significantly better than existing CNN architectures, particularly on the first subset. The MUSCLE Net further improved it by removing more false positives while maintaining true positives. In each column, the bold number indicates the most significant performance. The comparisons are among small and large groups, respectively. The detection rates of subjects with large lesions are all 100% so no bold numbers are identified.

		Dice	m#FP	m#FN	mSFP	mSFN	DR
DeepLab without CRF (Chen et al., 2014)	Small	0.39	12.84	4.96	**8.16**	3.52	0.90
	Large	0.75	12.72	6.00	**8.52**	5.80	1.00
FCN (Long et al., 2015)	Small	0.41	16.74	3.63	9.81	**3.16**	0.92
	Large	0.77	15.56	4.62	10.23	5.16	1.00
U-Net (Ronneberger et al., 2015)	Small	0.43	12.81	3.80	11.75	3.61	0.92
	Large	0.79	12.97	**4.56**	10.73	5.87	1.00
DeconvNet (Noh et al., 2015)	Small	0.47	11.38	3.75	10.21	3.21	0.92
	Large	0.79	12.98	4.72	9.92	**4.72**	1.00
EDD Net	Small	0.56	5.58	**3.58**	10.59	3.17	0.92
	Large	**0.83**	6.82	**4.56**	10.38	5.06	1.00
EDD + MUSCLE Net	Small	**0.61**	**2.97**	3.83	12.58	3.68	**0.93**
	Large	**0.83**	**4.16**	4.78	10.90	5.58	1.00

### 5.6 Running time

The pre-processing computation was run on a desktop PC, which is an HP Elite 8300, with an i7 processor and 16 GB RAM. The CNNs were trained and tested on an NVIDIA Tesla K80 GPU processor. We tested the running time of each stage of our proposed pipeline and the results were shown in Table 6. In summary, to test a new DWI scan, it costs less than 1 s, which is very fast.

**Table 6:** Running time of our proposed pipeline. The unit of time in testing is second and it in training is hour. The numbers in testing are in the form of mean ± std while the training time was measured in once.

	Running time	
	Testing (s)	Training (h)
Pre-processing	0.20 ± 0.10	–
EDD Net	0.63 ± 0.07	26.61
MUSCLE Net	0.07 ± 0.05	0.11
Total	0.90 ± 0.12	26.72

## 6 Discussion and conclusion

In this paper, we have presented a novel framework based on deep CNNs to segment the acute ischemic lesions in DWI. To the best of our knowledge, it is the first fully automatic method developed for this problem. The algorithm is validated on a large real clinical dataset and achieves the state-of-the-art, which is 0.67 in terms of the Dice coefficient in average. Several visual examples of the segmentation results are shown in Fig. 8.

Although the combination of EDD + MUSCLE Nets achieves very good results, the proposed approach still has a few limitations: First, semantic segmentation of objects in images in multiple scales remain a challenge that it is not fundamentally solved. Second, the training and testing is not end-to-end, which decreases the system's efficiency. Finally, in the second stage, we only consider the false positives. However, there are still a small number of false negatives which must be corrected.

In the future, further improvements could be achieved in several aspects. In particular, more DW images should be collected for training and testing. Our method is capable of automatically generating acute ischemic lesion segmentations. Experts could create the manual annotations based on the automatic segmentations, which will be less expensive in terms of time and effort. In addition, the framework could be adapted so that the end-to-end training is possible. Last but not least, convolutions in our proposed networks could be extended to 3D, which may reduce more false positives. 3D convolutions require the image patches and/or volumes to be isotropic in 3D (Kamnitsas et al., 2015, Kamnitsas et al., 2016b). However, image slices in our dataset are very thick and simple processes such as resampling cannot provide satisfactory results. Therefore, we consider to employ image super resolution techniques (Oktay et al., 2016) to enhance the images in 3D. Then 3D convolutions can be used in our CNNs.
